# Determination of the androgen receptor status of disseminated tumor cells in primary breast cancer patients

**DOI:** 10.1007/s00404-023-07225-z

**Published:** 2023-10-30

**Authors:** Natalia Krawczyk, Bernadette Jaeger, Piperek-Jäger Martina, Lopez-Cotarelo Rodriguez-Noriega Cristina, Neubacher Melissa, Banys-Paluchowski Maggie, Meier-Stiegen Franziska, Neubauer Hans, Niederacher Dieter, Ruckhäberle Eugen, Mohrmann Svjetlana, Hoffmann Jürgen, Kaleta Thomas, Esposito Irene, Fehm Tanja

**Affiliations:** 1 Department of Obstetrics and Gynaecology, University of Duesseldorf, Moorenstr. 5, 40225 Duesseldorf, Germany; 2 Department of Pathology, University of Mannheim, Theodor-Kutzer-Ufer 1-3, 68167 Mannheim, Germany; 3 Department of Obstetrics and Gynecology, University Hospital of Schleswig Holstein, Campus Lübeck, 23538 Lübeck, Germany

**Keywords:** Predictive marker, Minimal residual disease, Targeted therapy

## Abstract

**Purpose:**

Androgen receptor (AR) can serve as a new therapeutic target since it was shown to play a proliferative role in several breast cancer (BC) subtypes. Moreover, AR positivity has been suggested to reflect the metastatic potential of tumor cells in some BC subtypes. The aim of this study was to determine the AR expression on disseminated tumor cells (DTCs) as a surrogate marker of minimal residual disease (MRD) and potential precursor of metastasis in early BC.

**Methods:**

Bone marrow (BM) aspirates from 62 DTC-positive early BC patients were included into this study and analyzed by immunofluorescence staining for the presence of AR-positive DTCs. CK-positive, CD45-negative cells containing an intact nucleus (DAPI positive) were identified as DTCs. AR expression of the primary tumor (PT) was assessed by immunohistochemistry on formalin-fixed, paraffin-embedded (FFPE) tumor sections from core biopsies and surgical specimens.

**Results:**

AR status of DTCs could be determined in 21 patients. We detected AR-positive DTCs in nine samples (43%). AR expression of DTCs and corresponding PT showed a concordance rate of 33%. The DTC-AR status did not correlate with clinicopathological factors, nor did we observe a significant correlation between the AR status of the PT and other established prognostic factors for BC.

**Conclusion:**

AR-positive DTCs can be detected in BM of early BC patients with a marked discordance of the AR status between DTCs and corresponding PTs. The clinical significance of these findings needs further investigation.

## What does this study add to the clinical work

**Table:** 

We demonstrate the feasibility of assessing the AR status on DTCs using immunofluorescence staining and we show that a relevant proportion of DTCs express AR. Similar to other hormone receptors in breast cancer, we describe a discrepancy between the AR status of DTCs and the corresponding PT.	

## Introduction

The androgen receptor (AR) belongs to the nuclear steroid receptor family. As a ligand-dependent transcription factor, the AR in its unbound state is located in the cytoplasm. Upon binding, it undergoes dimerization and translocates to the nucleus where it binds its target genes [1–3]. Even though AR is expressed in over 70% of estrogen receptor (ER)-positive and in up to 45% of triple-negative breast cancer (TNBC), its role in breast cancer (BC) has not been completely elucidated. Its prognostic value was shown to differ between intrinsic BC subtypes [4–11]. In ERα-positive BC cells, the AR exerts either proliferative or anti-proliferative activity depending on co-expressed ERα level and the availability of the respective ligand [12–14]. In addition, overexpression of AR in ERα-positive BC cells seems to be involved in one of the resistance mechanisms to tamoxifen, demonstrated to be reversed by the treatment with AR antagonists [15]. Interestingly, most studies investigating the clinical relevance of AR positivity in ER-positive BC point toward a favorable prognosis [16, 17]. However, others report no prognostic impact of AR expression in this BC subtype [18].

Simultaneously, there is consensus on the proliferative activity of AR in HER2 + BC and TNBC, in which anti-androgen treatment can inhibit cell growth [19, 20]. Moreover, AR expression is increased in TNBC in early metastatic lesions compared to the primary tumor (PT) suggesting that AR supports tumor cell survival metastatic spreads [21]. Based on these data, AR represents a potential therapeutic target in all BC subtypes, which is why it is currently being investigated in several clinical trials for early and metastatic disease (see supplementary Tab. S1).

Disseminated tumor cells (DTCs) in bone marrow (BM) are presumed to be a surrogate marker for minimal residual disease (MRD) in BC patients. Their detection at diagnosis as well as after completion of cytotoxic treatment is associated with an impaired outcome [27–29]. However, not all patients with detactable DTCs will suffer from relapse, supporting the hypothesis of metastatic inefficiency of these cells [27]. Therefore, efforts have been made to further characterize the metastatic potential of DTCs and their role as a predictive therapeutic marker [30–33].

Because AR represents a potential therapeutic target in BC and AR positivity may reflect the metastatic potential of tumor cells in some of BC subtypes, the aim of our study was to evaluate the AR status of DTCs in a cohort of early BC patients and compare it with the AR status of the PT.

## Methods

### Patient cohort

**Fig. 1:**
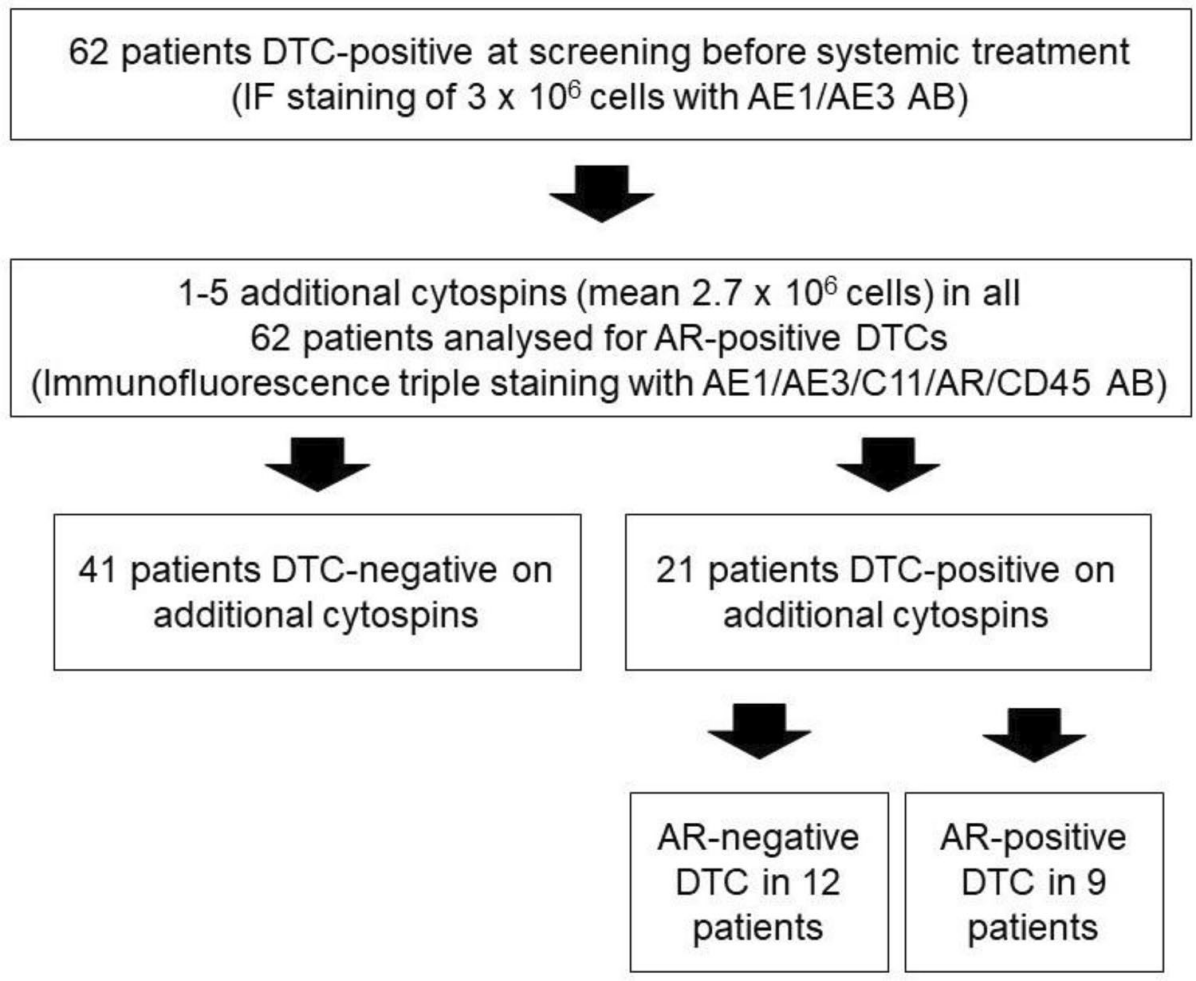
Study flow chart. DTC: disseminated tumor cell, AB: antibody, IF: immunofluorescence

Sixty-two DTC-positive patients with intraoperative bone marrow aspiration prior systemic therapy, treated from 2014 to 2019 at the Department of Obstetrics and Gynecology, University of Duesseldorf, were included in the study. Fifty-four patients (78%) were treated adjuvant, whereas neoadjuvant chemotherapy was planned in eight patients (13%) (Fig. 1, Table 2). This study was approved by the ethical committee of Heinrich Heine University in Duesseldorf (2018–56-FmB).

## Collection and analysis of bone marrow

Between 10 and 15 ml of BM were aspirated intraoperatively from the anterior iliac crest under general anesthesia and processed within 24 h. All samples were collected after patients gave written informed consent. BM samples were separated by density centrifugation over Ficoll (Biochrom, Germany) with a density of 1.077 g/ml. If necessary, red blood cells were lysed with lysis buffer (155 mM NH_4_Cl, 10 mM KHC0_3_, 0.1 mM EDTA pH 7.2). Using a cytocentrifuge (Hettich, Tuttlingen, Germany), 1.5 × 10^6^ mononuclear cells were spun onto a glass slide and dried at room temperature overnight. For each patient, two slides (3 × 10^6^ cells) were analyzed for the presence of DTCs and the remaining slides were stored at −20 °C. Slides were then fixed in a 4% neutral buffered formalin solution for 10 min and were rinsed in phosphate-buffered saline. Immunofluorescence staining was performed using eFluor™ 615 conjugated monoclonal mouse AE1/AE3 Pan Cytokeratin Antibody (Thermo Fisher Scientific) according to the manufacturers' instructions. Slides were then manually evaluated based on the morphological criteria of the International Society of Hematotherapy and Graft Engineering Working group for standardization of tumor cell detection and the consensus statements [34, 35]. CK-positive, CD45-negative cells with an intact nucleus (DAPI positive) were identified as DTCs. In the subset of initially DTC-positive patients (*n* = 62), between one to five additional slides per patient (mean 1.8 ± SD 0.686) were analyzed by immunofluorescence triple staining for the presence of AR-positive DTCs. Heterogeneous distribution of cells on a cytospin and freezing conditions might lead to loss of DTCs, and thus differences in positivity. Therefore, DTCs on additional cytospins could only be detected in 21 of the original 62 DTC-positive patients, 41 then had to be classified as DTC negative and could not be used for further analysis (Fig. 1). For positive control AR-positive LNCaP cells and for negative control AR-negative Du145 cells were mixed with peripheral blood mononuclear cells (PBMCs) from a healthy volunteer. The cytospins were prepared, stored, and fixed in the same way as described for the patient samples.

### Immunofluorescence staining

Additional slides were thawed at room temperature in a humid chamber for 20 min (in 18 patients, 1 additional slide was analyzed; in 42 patients, 2 slides and 5 slides in 2 of our initially DTC-positive patients). After an initial wash step with PBS (Sigma, Munich, Germany), cells were permeabilized with PBS containing 0.1% Triton X-100 for a period of 5 min prior to blocking with Protein Block Solution (DAKO, CA, USA) for another 10 min. A triple immunofluorescent staining, established for circulating tumor cells (CTCs) from peripheral blood, was used as described previously by our group with minor modifications [36]. The AR staining was performed using the AR (D6F11) XP rabbit monoclonal antibody (1:50, Cell Signaling Technologies Inc., Danvers¸ MA, USA), the pan-cytokeratin (CK) antibody (CK all C11 AE1/AE3) directly conjugated to Tetramethylrhodamine (TRITC) (1: 40, Aczon, Bologna, Italy) and an Alexa Fluor 647-conjugated CD45 antibody (35-Z6) (1:20, Santa Cruz Biotechnology, Dallas, TX, USA) for 45 min. Cytospins were subsequently incubated with 4′6-diamidino-2-phenylindole (DAPI) (Thermo Fisher Scientific, Waltham, USA) for nuclear DNA staining and a secondary donkey anti-rabbit antibody, labeled with Alexa Fluor 488 (1:500, Invitrogen by Thermo Fisher Scientific, Carlsbad, USA) for 30 min. Samples were mounted using Dako Fluorescent Mounting Medium (Dako, Carpinteria, USA). The control slides of Du145/PBMC (negative control) and LNCaP/PBMC mixtures (positive control) were included in each batch of samples.

**Fig. 2:**
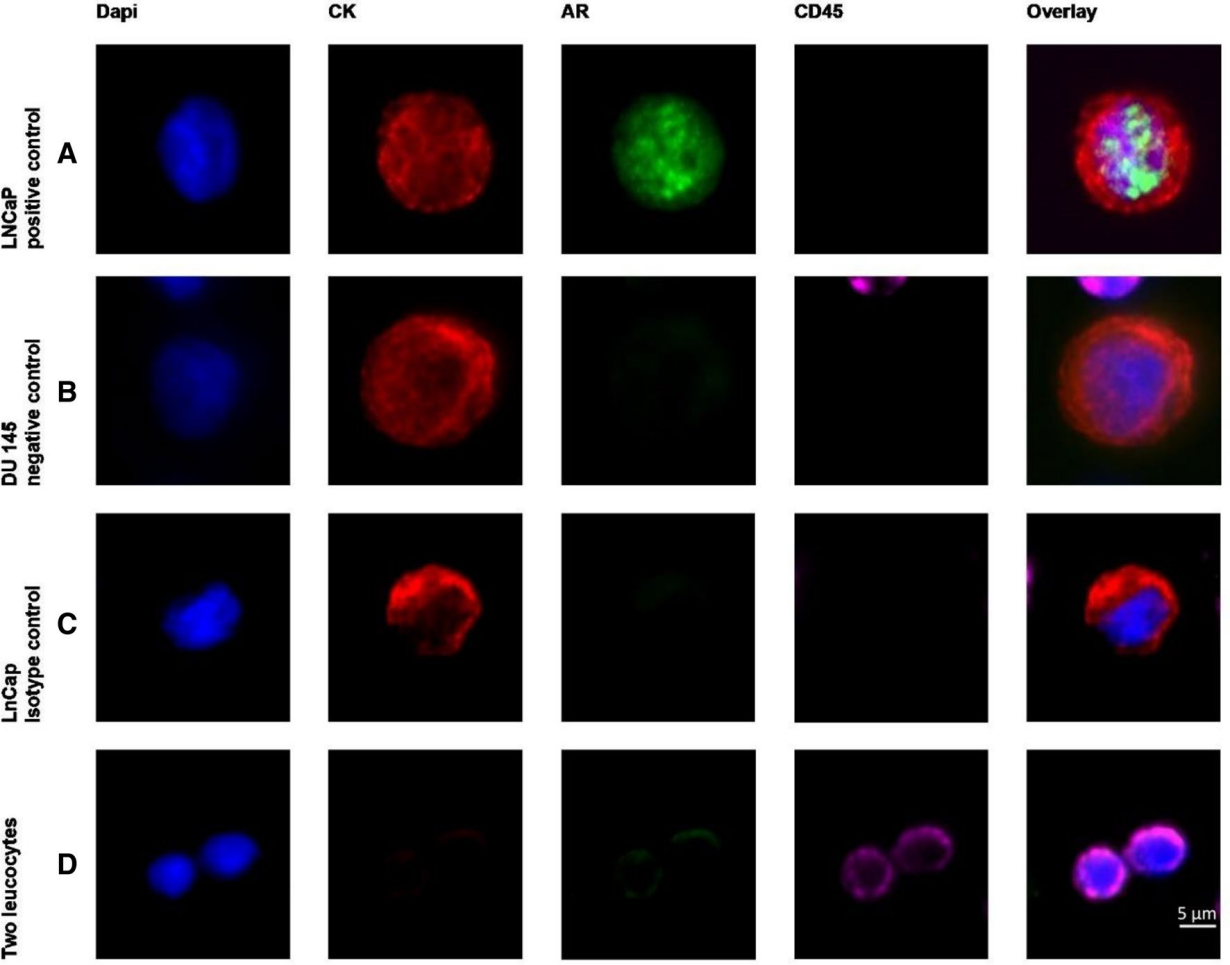
Androgen receptor (AR) control stainings: **A** LNCaP prostate cancer cell line (positive control), **B** Du145 prostate cancer cell line (negative control), **C** AR isotype control staining (LNCaP), **D** CD45 positive control staining (leucocyte)

Slides were manually analyzed for the presence of tumor cells using a computerized fluorescence microscope Axioplan 2 (× 40 immersion objectives, Carl Zeiss Micro Imaging GmbH, Göttingen, Germany). To screen for AR-positive DTCs, a single-pass filter for individual fluorochromes, FITC, Texas Red or DAPI, and a triple-pass filter for FITC/TRITC/DAPI were used. Immunostained cells were evaluated based on the criteria described above. DTCs with either moderate or intense AR staining were considered AR positive. Slides were evaluated by two, or in doubtful cases three, independent investigators (MP, NK, and HN). Positive and negative control stainings are shown in Fig. 2.

### Immunohistochemistry

FFPE tumor sections from core biopsies or surgical resection were used for immunohistochemical detection. Immunohistochemistry was performed on a Ventana BenchMark ULTRA staining instrument (Roche, Mannheim, Germany) according to the manufacturer´s instructions. FFPE tumor tissues were sectioned at a thickness of 4 to 5 μm and stained with an anti-AR antibody (1:30 dilution, clone F39.4.1, BioGenex, San Ramon, USA). Detection was performed with the OptiView DAB IHC Detection Kit (Roche, Mannheim, Germany), following the manufacturer´s instructions.

AR expression was quantified based on the percentage of positive cells in at least 500 neoplastic cells counted in the tumor area. An unequivocal immunohistochemical staining was defined as a positive nuclear staining, which was clearly visible with the use of a 2 × or 5 × objective. Negative staining was defined as absence of nuclear expression. An unequivocal staining in > 1% tumor cells was considered AR positive.

### Statistical analysis

A Chi-squared test was used to evaluate the relation between AR-positive DTCs and/or primary tumor and clinicopathological factors. Statistical analysis was performed by SPSS, version 25 (SPSS Inc., Chicago, IL, USA). Values of* p* < 0.05 were considered statistically significant.

## Results

### Patients’ characteristics

BM aspirates from 62 primary BC patients, 60 females and 2 males, were eligible for this study. Fifty patients (81%) were diagnosed with a hormone receptor (HR) + /HER2-negative tumor, six cases (10%) with had immunohistochemistry staining indicating HR + /HER2 + tumor as evaluated by immunhistochemistry, and five patients (8%) had a TNBC (Table 1).

**Table 1:** Clinical data of included patients

	*n* (%)
Total	62
Menopausal status	
Premenopausal	21 (34)
Postmenopausal	39 (63)
Male	2 (3)
Treatment	
Adjuvant	54 (78)
Neoadjuvant	8 (13)
Tumor size*	
T1	30 (48)
T2–4	32 (52)
Nodal status*	
Negative	40 (65)
Positive	20 (32)
Unknown	2(3)
Histology	
NST	51 (83)
Lobular	9(15)
Other	2 (2)
Grading	
I	4 (7)
II	43 (69)
III	15 (24)
ER status	
Negative	6 (10)
Positive	56 (90)
PR status	
Negative	12 (19)
Positive	50 (81)
HER2 status	
Negative	54 (87)
Positive	8 (13)
IHC subtype	
HR + /HER2-	50 (81)
HR + /HER2 +	6 (10)
HR-/HER2 +	1 (2)
TNBC	5 (8)

ER = estrogen receptor. HER2 = human epidermal growth factor receptor 2. PR = progesterone receptor. IHC = immunohistochemistry. TNBC = triple-negative breast cancer

### DTC detection and AR expression on DTCs

**Fig. 3:**
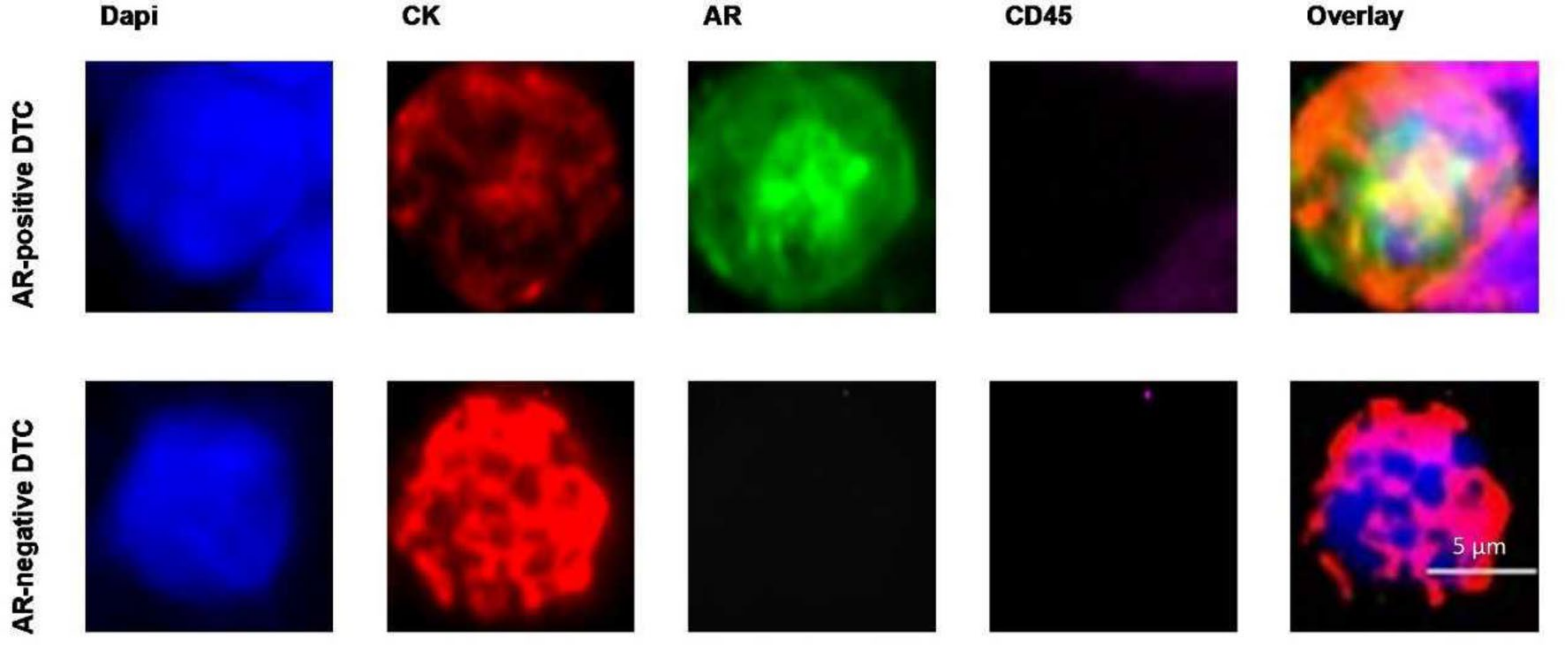
Androgen receptor (AR) staining of DTCs in primary breast cancer patients: **a** AR-positive staining. **b** AR-negative staining

In 21 of the 62 initially DTC-positive patients (34%), analysis of additional cytospins detected at least 1 DTC in the BM. The DTC count ranged from 1 to 20 cells. However, 19 of the 21 further DTC-positive patients (90%) had only 1 DTC in BM. In 9 out of the 21 further DTC-positive patients (43%), AR-positive DTCs could be detected. We detected no correlation between AR status of DTC and any of the clinicopathological factors (Table 2). AR staining of DTCs in BC patients is depicted in Fig. 3.

**Table 2:** Clinical data of 21 patients included in further analysis of AR status of DTC

	*N* (%)	AR-positive DTCs (%)	*p *value*
Total	21 (100)	9 (43)	
Menopausal status			0.58
Premenopausal	8 (38)	3 (37)	
Postmenopausal	12 (57)	6 (50)	
Male	1 (5)	0 (0)	
Treatment			0.72
Adjuvant	18 (86)	8 (44)	
Neoadjuvant	3 (14)	1 (33)	
Tumor size*			0.71
T1	11 (52)	5 (46)	
T2–4	10 (48)	4 (40)	
Nodal status*			0.88
Negative	16 (76)	7 (44)	
Positive	5 (24)	2 (40)	
Unknown	0(0)	0 (0)	
Histology			0.10
NST	18 (86)	9 (50)	
Lobular	3 (14)	0 (0)	
Other	0 (0)	0 (0)	
Grading			0.17
I	2 (9.5)	2 (100)	
II	13 (62)	4 (31)	
III	6 (28.5)	3 (50)	
ER status			0.09
Negative	2 (9.5)	2 (100)	
Positive	19 (90.5)	7 (37)	
PR status			0.75
Negative	4 (19)	2 (50)	
Positive	17 (81)	7 (41)	
HER2 status			0.10
Negative	18 (86)	9 (50)	
Positive	3 (14)	0 (0)	
IHC subtype			0.085
HR + /HER2-	16 (76)	7 (44)	
HR + /HER2 +	3 (14)	0 (0)	
HR-/HER2 +	0 (0)	0 (0)	
TNBC	2 (10)	2 (100)	

ER = estrogen receptor. HER2 = human epidermal growth factor receptor 2. PR = progesterone receptor. IHC = immunohistochemistry. TNBC = triple-negative breast cancer

*By Chi-squared test

### AR status of PT

We determined the AR status of the PR in the 21 DTC-positive patients. Tumors were AR positive in 13 patients (62%); 12 out of 16 HR + /HER2− (75%), 1 out of 3 HR + /HER2 + (33,3%) patients, and none out of 2 TNBC patients, respectively. No significant correlation could be observed between the AR status of the PT and other established prognostic factors. AR status of DTCs and corresponding PTs showed a concordance rate of 33% (Table 3).

**Table 3:** Androgen receptor (AR) status of DTC and corresponding primary tumor (PT)

AR status		PT		Total (%)
		AR negative (%)	AR positive (%)	
DTC				
AR negative (%)		3 (14)	9 (43)	12 (57)
AR positive (%)		5 (24)	4 (19)	9 (43)
Total (%)		8 (38)	13 (62)	21 (100)

## Discussion

AR is considered a prognostic factor and potential predictive biomarker in BC, even though its role has not been fully understood. This may be due to biological or technical reasons. As for the biological aspects, several studies indicate that its role depends on the relative concentrations of circulating estrogens, androgens, and on the tumor microenvironment [37]. Therefore, increasing evidence suggests a differential function of AR among intrinsic subtypes of BC. In TNBC and HER2 + BC, AR expression increased tumor proliferation, while in ERα-positive BC, its role is ambiguous with either pro- or anti-proliferative effects, depending on the level of co-expressed ERα and the presence of the particular ligand [12–14, 38]. Out of the two male patients included in the study, one was DTC positive on the additional cytospins and could be analyzed further. Regarding the AR status, we found a discrepancy (AR-negative DTCs while AR-positive PT). Little is known about AR status in male breast cancer, but it is receiving increasing attention here as an additional therapeutic target. However, it is not clear whether the findings obtained in female breast cancer can be readily applied to male breast cancer. Mutational and epigenetic similarities and differences may play a role in this regard [39]. In addition, the ratio of AR expression to other HR such as the progesterone receptor or ER may provide clues to the efficacy of endocrine therapy [40] or the clinical outcome [41]. Moreover, it has been suggested that AR expression status reflects metastatic potential in BC [21]. Therefore, given the experience in prostate cancer, an interest in AR targeted treatment in all BC subtypes increased recently. Various AR targeting drugs, mostly anti-androgens and selective AR modulators, are currently being evaluated as treatment options of metastatic and early disease.

The technical debate regarding AR expression in BC does not only concern the detection method and the cutoff value, but also the tissue used. Detection of HR status in BC is a well-established element for stratification into different risk profiles and for deciding on potential treatment options. Immunohistochemistry is an easy-to-use, cost-effective and well-established method. However, there is no standardized protocol to assess AR status, and thresholds vary between different studies (1% this study, 10% (Ref) or 50% (Ref)) [42]. An H-score has also been proposed to integrate staining intensity and percentage of positive cells [42]. A standardization of this method is needed to be able to compare different studies and to pronounce therapy recommendations based on a reliable AR status. In addition, fluorescence in situ hybridization or PCR-based methods allow to determine genetic aberrations or gene expression levels in AR [43]. As a specimen, tumor tissue, either PT or metastatic lesion, is usually used to define AR status. However, differences in HR status between PT and metastatic lesions are a common phenomenon in BC due to tumor heterogeneity with different evolving tumor clones that may occur multiple times and usually represent disease worsening [44, 45]. Therapeutic pressure could be a reason for this development, also in the sense of acquiring therapy resistance [46]. Few studies have been performed on the AR status of the parental tumor tissues in BC patients. Differences in AR status between PT and lymph node metastases were described by Kraby et al. in 21.4%. In this study, the metastatic lesion in the lymph nodes was described as AR positive while the PT was AR negative [47].

Given a low overall concordance of AR expression in PT and metastasis, it is reasonable to assess the AR status on all available tumor specimens, before recommending anti-AR therapy [42]. Even though patient’s stratification for AR-targeted therapy is usually based on AR status of tumor tissue, efforts have been made to examine AR status using liquid biopsies. The ability to evaluate the AR status in serum, plasma, or urine as well as circulating tumor cells (CTCs) suggests the use of AR expression as a biomarker for disease monitoring in real time. This was demonstrated by the extensive studies on prostate cancer [42]. In BC patients, the main focus of liquid biopsy approaches is to determine the AR status of CTCs, reflecting the characteristics of the dominant cell population in metastasis. It can further serve as a surrogate of MRD and possible source of future metastases in the adjuvant setting [48]. AR status of CTCs in metastatic spreads and corresponding PT has been evaluated by two previous groups [49, 50]. Kruijff et al. 2019 evaluated AR status of CTCs and corresponding PT in 52 MBC patients showing a concordance rate of 42% (22/52 patients). Interestingly, most patients demonstrated AR-negative CTCs (29/44 patients (65%) with AR-positive PT and 7/8 patients (87.5%) with AR-negative PT, respectively) whereas only 1 out of 8 patients (12.5%) with AR-negative PT presented with AR-positive CTCs in PB [49]. In our study AR-positivity rate of DTCs was also lower than the AR-positivity rate of corresponding PT (43 vs. 62%, respectively). However, AR-negative PTs more often presented as AR-positive DTC (5/8 patients, 62.5% (Table 3)). Li et al. observed a concordant AR status of CTCs and PT tissue in 51 out of 75 MBC patients (68%) and 80% of analyzed patients presented with AR-positive CTCs (60/75). Interestingly, the AR positivity rate of CTCs was slightly higher than AR-positivity rate of PT (80% in CTCs vs. 77% in PT, respectively). The fact that AR-positivity rates of PTs in the Li et al. trial was higher than in the study by Kruijff et al. and our analysis may be caused by different clinical features, such as the fact that all tumors analyzed by Li et al. were defined as HR + /HER2-negative, compared to 81% in our study and 63% in the trial by Kruijff et al.. A HR + /HER2-negative tumor biology was previously reported to correlate with AR positivity by up to 90% [11, 38, 49, 50]. Another reason for the increased AR-positivity rate in CTCs comparing to PT observed by Li et al., may be that all patients in this trial were pretreated with nonsteroidal aromatase inhibitors, and aromatase inhibitor treatment was previously shown to increase AR activity in BC [51]. Aceto et al. found a correlation of the duration of endocrine therapy and AR expressing CTCs. Further, the active splice variant AR-v7, as detected by RNA sequencing, correlated with the presence of bone metastasis [52]. Even though AR expression might be a possible target for anti-androgenic intervention also in BC patients, it seems to interfere with aromatase inhibitor therapy and to trigger endocrine resistance mechanism. Activated pathways identified by single-cell RNA sequencing in CTCs from patients with ER + MBC include the AR pathway, particularly in bone rather than visceral metastases. In the context of a comprehensive picture of the AR expression of each tumor under consideration of its genetic heterogeneity, it is of interest to assess the AR status also of DTCs in BM as a surrogate marker of MRD and a potential source of future metastases. While data comparing AR expression on DTCs and matching PT are missing, our study shows DTCs in BM of early BC patients heterogeneously express AR and that AR-expression status of DTCs and PT differ. In summary, BM aspirates from 62 early BC patients were analyzed by triple immunofluorescence staining in terms of AR expression on DTCs prior to any systemic treatment. In 21 of 62 patients (34%), AR status of DTC could be determinate and in 9 out of 21 patients (43%), at least one AR-positive DTC has been detected. This is, to the best of our knowledge, the first trial evaluating AR expression on DTCs in early BC. In addition, we assessed the AR status of PT in these 21 DTC-positive patients and compared it with AR status of DTCs in BM, demonstrating a concordance rate of 33% (Table 3).

Phenotypic discrepancies between PT and DTCs in BM of early BC patients with regard to predictive and prognostic factors, e.g., HER2 or ER status, is a phenomenon described previously by our group and others [30–32, 53, 54]. These analyses demonstrated DTCs to be often HER2 positive, despite HER2-negative PT and ER negative in patients with ER-positive PT reflecting their rather aggressive phenotype. In our analysis, 12 of 21 DTC-positive patients (57%) were identified with AR-negative DTCs.

Although this is the first study to investigate AR expression on DTCs and to compare it with the AR status of PT tissue, several limitations must be mentioned. The patient collective was selected retrospectively, without clinical consequences drawn from the AR status and without the possibility of collecting follow-up data. In addition, our patient collective is rather small with a low positivity rate of 9/21 AR-positive DTCs. Technically, different staining methods (IHC vs. Immunofluorescence) and different AR antibodies have been used for the AR staining of PT and DTC, respectively.

Detection of AR status on DTCs is possible, as is detection of AR status of CTCs. Comparison of AR status, similar to comparison of ER or HER2 status of MRD with tumor tissue (PT or metastatic lesion) can reveal differences and might contribute to selected patients for anti-androgen therapy. Whether these findings can be implemented in daily clinical routine needs further evaluation. Targeted therapies based on MRD findings are conceivable, which then serve as liquid biopsies and allow regular therapy monitoring. In this context, however, CTCs or even cell-free DNA might be easier to determine for both patients and physicians.

## Data Availability

The data underlying the results of our study in the manuscript are available from the corresponding author upon request.

## Abbreviations

AR: Androgen receptor
BC: Breast cancer
BM: Bone marrow
CK: Cytokeratin
CTC: Circulating tumor cell
DAPI: 4′6-Diamidino-2-phenylindole
DTC: Disseminated tumor cell
EpCAM: Epithelial cell adhesion molecule
ER: Estrogen receptor
FFPE: Formalin-fixed, paraffin-embedded
FITC: Fluorescein isothiocyanate
HR: Hormone receptor
MRD: Minimal residual disease
PB: Peripheral blood
PBMC: Peripheral blood mononuclear cells
PT: Primary tumor
TNBC: Triple-negative breast cancer
